# Prosodic focus marking in Seoul Korean-speaking children: the use of prosodic phrasing

**DOI:** 10.3389/fpsyg.2024.1352280

**Published:** 2024-07-31

**Authors:** Anqi Yang, Taehong Cho, Sahyang Kim, Aoju Chen

**Affiliations:** ^1^School of Foreign Languages, Tianjin University, Tianjin, China; ^2^Hanyang Institute for Phonetics and Cognitive Sciences of Language, Hanyang University, Seoul, Republic of Korea; ^3^Department of English Education, Hongik University, Seoul, Republic of Korea; ^4^Institute for Language Sciences, Utrecht University, Utrecht, Netherlands

**Keywords:** acquisition, focus, prosodic phrasing, prosodic marking of focus, Seoul Korean

## Abstract

**Introduction:**

Prosodic focus marking in Seoul Korean is known to be achieved primarily through prosodic phrasing, different from the use of prosody for this purpose in many other languages. This study investigates how children use prosodic phrasing for focus-marking purposes in Seoul Korean, compared to adults.

**Methods:**

Using a picture-matching game, we elicited semi-spontaneous production of SOV sentences in various focus conditions from monolingual Seoul Korean-speaking children aged 4 to 11 years.

**Results:**

We found that the children varied prosodic boundaries to distinguish narrow focus from pre-focus and broad focus in a largely adult-like manner at the age of 4 to 5; at this age, they did not distinguish narrow focus from post-focus or contrastive focus using prosodic boundaries, similar to the adults. Their use of the prosodic boundaries in distinguishing the focus conditions was not fully adult-like in terms of frequency until the age of 10 to 11.

**Discussion:**

In conjunction with the findings of previous studies on the acquisition of focus marking in Germanic languages, performed using a similar experimental method, our findings suggest that Seoul Korean-speaking children acquire the use of prosodic phrasing earlier than Dutch-speaking children acquiring the use of pitch accent but slightly later than Stockholm Swedish-speaking children acquiring the use of a prominence-marking high tone. These findings imply that the rate of focus-marking acquisition depends on the transparency of the form-meaning mapping between the phonological cue and focus.

## 1 Introduction

Previous studies on the acquisition of prosodic focus marking in various languages have revealed that children acquiring a language that relies on prosody or both prosody and syntax for focus marking show adult-like use of at least some language-specific prosodic focus-marking cues by the age of 5 and further develop this ability until the age of 10 or 11 (e.g., Hornby and Hass, 1970; MacWhinney and Bates, 1978; Wells et al., 2004; Thorson and Morgan, 2020, on English; Chen, 2009, 2011a,b; Romøren, 2016, on Dutch; Grünloh et al., 2015, on German; Romøren and Chen, 2021, on Stockholm Swedish, hereafter Swedish; Yang and Chen, 2018, on Mandarin). However, the specific developmental trajectory differs for children acquiring different languages due to typological differences in prosodic system and prosodic focus marking (Chen, 2018). The present study is concerned with children learning Seoul Korean (hereafter Korean), a language that differs from more widely studied languages such as Dutch, English, Finnish, German, Swedish, and Mandarin in that it primarily uses prosodic phrasing in focus marking (Jun, 1993, 2007b; Jun and Lee, 1998).

Focus is prosodically encoded in many languages (e.g., Vallduví and Engdahl, 1996; Gussenhoven, 2007; Kügler and Calhoun, 2021). Among the most frequently studied focus types in literature, narrow focus (i.e., focus on a word of a syntactic constituent, like “the bread” in (1B)) differs from narrow contrastive focus (i.e., narrow focus carrying contrast^1^, hereafter contrastive focus, like “the bread” in (2B)) in contrastivity, and differs from broad focus (i.e., focus over a constituent larger than a word, like (3B)) in size of focal constituent. Generally, a word is realized with more prosodic prominence in narrow focus and contrastive focus than when not focused or in broad focus. Contrastive focus can be realized with additional prosodic prominence, compared to non-contrastive narrow focus, in certain speech styles in some languages (e.g., read speech in Mandarin) (Chen and Braun, 2006).

1. A: Look! The dog^2^, and it holds a painting brush. It looks like the dog draws something. What does the dog draw?
  B: 개가    [빵을]^3^    그려요.
  kɛka    p*aŋɨl    kɨljʌjo.
  The dog    [the bread]    draws.
2. A: Look! The bear. The bear looks a bit puzzled. It looks like the bear looks for something. I will make a guess: The bear looks for the egg.
  B: 곰이    [빵을]    찾아요.
  komi    p*aŋɨl    tʃʰatʃajo.
  The bear    [the bread]    looks for.
3. A: Look! My picture is very blurry. I cannot see anything clearly. What happens in your picture?
  B: [말이    빵을    그려요].
  mali    p*aŋɨl    kɨljʌjo.
  [The horse    the bread    draws].

Regarding the precise prosodic means for achieving prominence, we distinguish *phonetic implementation (hereafter phonetic means)* and *phonological means*, following Chen (2009, 2011b, 2018). Specifically, some languages (e.g., Mandarin and Cantonese) rely on phonetic means. Speakers of these languages vary the phonetic implementation of phonological categories such as lexical tones in the dimensions of duration, pitch, and intensity for focus-marking purposes, without changing the tonal identity of relevant words (e.g., Xu, 1999, on Mandarin; Wu and Xu, 2010, on Cantonese). For example, in Mandarin, a word in narrow focus is produced with a longer duration, wider pitch span, and higher intensity than the same word in non-focus, while its tonal category remains intact (e.g., Shih, 1988; Xu, 1999). Other languages (e.g., English, German, Dutch, Swedish, and Korean) primarily use *phonological means* to realize prosodic prominence. That is, speakers make coarse-grained changes in duration, pitch, and intensity that lead to a change in the phonological category of prosody. For example, in English, German, and Dutch, speakers can either accent words with certain types of pitch accents (e.g., rising vs. falling) or not accent words to distinguish narrow focus from non-focus (e.g., Gussenhoven, 2004, 2007; Baumann et al., 2006; Hanssen et al., 2008; Chen, 2009, 2011b); in Swedish, speakers can either assign or not assign a prominence-marking high tone to the end of a word for this purpose (Bruce, 2007; Romøren and Chen, 2021). However, these languages can differ in the transparency of the form-meaning mapping between the phonological cue and focus, i.e., how consistent the mapping is. For example, in Swedish, only focused words are produced with a word-final high tone. The mapping between the placement of the prominence-marking high tone and focus is thus highly transparent. By contrast, in Dutch, both focused and non-focused words can be accented with the same type of pitch accent, e.g., a falling accent in sentence-initial subject-noun phrases and a downstepped falling accent in sentence-final object-noun phrases, regardless of focus status. There is thus no consistent or transparent mapping between accentuation and focus (Chen, 2018). In such cases, speakers vary the phonetic implementation of pitch accents to distinguish focus and non-focus (Chen, 2009).

Differences in the transparency of the form-meaning mapping between the phonological cue and focus can lead to differences in the rate of acquisition in prosodic focus marking across languages (Chen, 2018). Previous studies on children acquiring a West Germanic language, which is relatively less transparent as discussed above, have shown that while children can already use accentuation to mark focus by the age of 5, their choice of accent type is not fully adult-like until the age of 7 or 8 (e.g., Hornby and Hass, 1970; MacWhinney and Bates, 1978; Wells et al., 2004; Thorson and Morgan, 2020, on English; Grünloh et al., 2015, on German; Chen, 2011a,b, on Dutch). In contrast, in Swedish, which is more transparent in the form-meaning mapping, phonological focus-marking is acquired earlier (Romøren, 2016; Romøren and Chen, 2021). That is, Swedish-speaking children are by and large adult-like at the age of 4 or 5 in assigning a prominence-marking high tone to the end of the word in narrow focus and contrastive focus conditions. The earlier acquisition of phonological focus marking in Swedish than in the West Germanic languages has been attributed to the more transparent form-meaning mapping between the phonological cue and focus in Swedish (Chen, 2018).

In the present study, we aim to extend the current understanding of the effect of transparency of form-meaning mapping by investigating how Korean-speaking children acquire the use of the phonological cue for focus-marking purposes. Korean is different from the previously studied languages in both prosodic system and prosodic focus marking. Regarding prosodic system, Korean has no word-level use of prosody. It is often classified as an edge-prominence language (Jun, 2014). That is, pitch movement in this language is aligned to prosodic phrases (Jun, 1993, 1998, 2000, 2005, 2014). Accentual Phrase (AP) is the smallest unit carrying a phrasal tone sequence, THLH, with the initial tone (T) being realized as either a high tone (H) or a low tone (L) at the left edge, depending on the laryngeal feature of the initial segment of the AP. The second H tone is generally realized on the second syllable of an AP, but is sometimes realized on the third syllable when an AP is longer than four syllables; when an AP contains fewer than four syllables, one or both of the two middle tones may be undershot, with the choice of tones undershot varied across speakers, discourse contexts, and other linguistic factors (Jun, 2005). The final H tone is realized on the last syllable and marks the right edge of an AP. In addition to the tonal marking at both edges, AP is also marked by domain-initial (segmental) strengthening at the left edge (Cho, 2022; Kim et al., 2024). An AP consists of one or more Phonological Words (PWs). An Intonational Phrase (IP) consists of one or more APs and is marked by a phrase-final boundary tone and phrase-final lengthening (or pre-boundary lengthening) at the right edge (Cho, 2022; Kim et al., 2024).^4^

Regarding prosodic focus marking, past work based on read speech shows that Korean primarily uses prosodic phrasing for this purpose (e.g., Jun, 1993, 2007b). A word in a narrow focus or contrastive focus condition typically initiates a prosodic phrase, which can be either an AP or an IP, with the following words tending to be integrated into the same phrase as the focused word, resulting in dephrasing (Jun, 1993; Jun and Lee, 1998; Oh, 1999; Jun and Kim, 2007; Jeon and Nolan, 2017). Given that a phrasal boundary is either present or not (i.e., a discrete concept), the use of prosodic phrasing can be considered a phonological means of focus marking, like accent placement and choice of accent type in Dutch and the placement of a prominence-marking high tone in Swedish. However, prosodic phrasing, including dephrasing, can be influenced by factors other than focus marking, such as speech rate, rhythm, semantic weight, length and syntactic structure of the utterance (*cf.* Jun, 1993, 2011). For example, when a short syntactic phrase (e.g., a verb phrase or noun phrase) or a short sentence as a whole is focused, each word in it tends to form an AP (Jun et al., 2006; Kim et al., 2006; Jun and Kim, 2007; Jun, 2011). Dephrasing does not play a role in focus marking in this case. Hatcher et al. (2023) have also demonstrated instances where focus realization can occur phrase-medially in Korean, notably without resorting to phrasing. The transparency of the form-meaning mapping between phonological cues and focus conditions in Korean can thus be considered lower, compared to Swedish. It may not be different from that in a West Germanic language, like Dutch,^5^ because both phrasing (in Korean) and pitch accent (in a West Germanic language) can occur in non-focused contexts.

However, prosodic phrase boundaries, especially IP boundaries, appear to be relatively easy to perceive in continuous speech streams, compared to the perception of prosodic prominence associated with pitch accent. This has been shown to be the case of both linguistically trained adult listeners (e.g., Grice et al., 1996, on German; Jun et al., 2000, on Korean; Escudero et al., 2012, on Catalan) and naïve adult listeners with no prior linguistic knowledge presented with an unfamiliar language for the first time (Cole and Shattuck-Hufnagel, 2016; Cole et al., 2017; Bishop et al., 2020). Development literature suggests a very early ability to perceive and process prosodic phrase boundaries. For example, French-learning infants exhibit sensitivity to prosodic boundaries at birth (Christophe et al., 1994, 2001). Infants learning a West Germanic language change from relying on all possible cues in perception of major prosodic phrase boundaries to a subset of the cues between 4 and 8 months, partially reflecting the relative importance of the cues in the target language (Seidl, 2007; Johnson and Seidl, 2008; Seidl and Cristià, 2008). They exhibit adult-like processing of major prosodic phrase boundaries in the brain at 6 months (Holzgrefe-Lang et al., 2018). These findings suggest that a prosodic boundary may be a perceptually more recognizable cue to focus than accentuation, making the mapping between a prosodic phrase boundary and focus possibly more transparent in Korean than the mapping between accentuation and focus in a West Germanic language to young language learners. Notably, children appear to be similar in their development in the production of pitch accents in a West Germanic language and the production of AP pitch patterns and IP-final boundary tones in Korean, independent of the mapping with meaning like focus. For example, by the age of 2, children acquiring a West Germanic language can produce the core of an adult-like inventory of pitch accents (Chen et al., 2020); Korean-speaking children can produce certain AP pitch patterns and IP-final boundary tones (Jun, 2007a).

According to Chen’s (2018) cross-linguistic model of acquisition of prosodic focus marking, higher transparency of the form-meaning mapping between focus and the phonological cue will lead to a faster rate of acquisition in prosodic focus marking. If this holds, we hypothesize that Korean-speaking children will exhibit adult-like phonological focus marking in some or all aspects relatively later than their Swedish-speaking peers, who are adult-like at the age of 4 to 5 years, but earlier than their Dutch-speaking peers, who are not fully adult-like yet at the age of 7 to 8 years.

To test this hypothesis, we have examined how Korean-speaking children aged 4 to 11 vary prosodic phrasing to distinguish (1) Narrow focus from non-focus (Effect of focus); (2) Narrow focus from broad focus (Effect of focal constituent size); and (3) Narrow focus from contrastive (narrow) focus (Effect of contrastivity), in comparison to Korean-speaking adults. Regarding the effect of focus, we predict that Korean-speaking children will be fully adult-like at the age of 7 to 8 years or later in more frequently using an AP and/or IP boundary before a word and a PW boundary after it when the word is in the narrow focus condition than in the non-focus conditions (i.e., pre-focus or post-focus). Regarding the effect of focal constituent size, we predict that the children will be fully adult-like at the age of 7 to 8 years or later in frequently using an AP and/or IP boundary before a word in both the narrow and broad focus conditions, and in more frequently using a PW boundary after a word when it is under narrow focus than broad focus. Regarding the effect of contrastivity, as previous studies on Korean reveal no evidence for the use of prosodic phrasing to mark contrastivity and there is no language-independent reason to use prosodic phrasing to mark a contrast, we predict that Korean-speaking children will not vary prosodic boundaries to distinguish narrow focus from contrastive focus, like adults and this may be observable at the age of 4 to 5 years.

## 2 Method

### 2.1 Participants

Three groups of children participated in the experiment, including six 4- to 5-year-olds (average age: 5;3, range: 4;10–5;10), eight 7- to 8-year-olds (average age: 8;0, range: 7;4–8;10), and eight 10- to 11-year-olds (average age: 10;10, range: 10;3–11;11). They were recruited via Hanyang Institute for Phonetics and Cognitive Sciences of Language in Seoul, and came from diverse social-economic backgrounds. Twelve adults (six females and six males, average age: 24 years, range: 19–28 years) participated as a control group. They were students of Hanyang University at the time of testing. All participants spoke Seoul Korean as their native language and did not have any known language and/or cognitive impairment.

### 2.2 The picture-matching game

**Figure 1:**
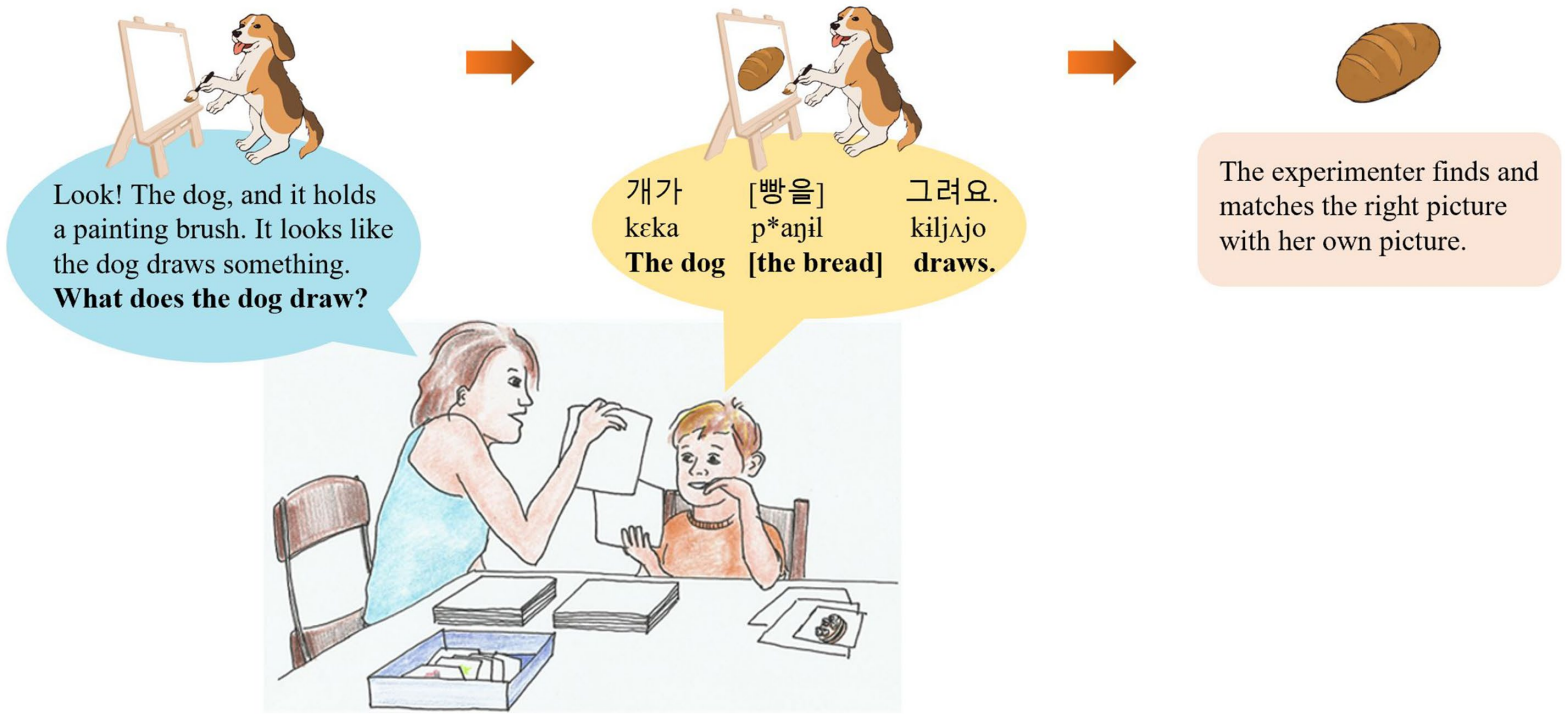
Illustration of the experimental setup.

We adapted the picture-matching game used in Chen (2011b) to elicit semi-spontaneous production of sentences. The same game was used in recent studies on prosodic focus marking in children acquiring other languages (Romøren, 2016; Liu, 2017; Yang and Chen, 2018; Romøren and Chen, 2021). In this game, the child was supposed to help the experimenter put pictures in matched pairs (Figure 1). Three piles of pictures were used. The experimenter and the child each held a pile of pictures. The third pile lay on the table in a seemingly messy fashion. The experimenter’s pictures always missed some information (e.g., the subject, the action, the object, or all three). The child’s pictures always contained all three pieces of information. On each trial, the experimenter showed one of her pictures to the child, described the picture and asked a question about it or made a remark about the missing information (in the contrastive focus condition). The child then took a look at the corresponding picture in his/her pile and responded to the experimenter’s question or remark. The experimenter then looked for the right picture in the messy pile and matched it with her own picture to form a pair.

As rules of the game, the child was asked to answer the experimenter’s questions in full sentences and not to reveal his/her pictures to the experimenter. We constructed an experiment protocol outlining each step of the game, specifying the experimenter’s instructions and responses for each trial. This protocol ensured consistent conduct of the experiment for all children and the provision of sufficient background information before each question or remark. The experimenter was instructed to follow the protocol closely but was encouraged to make spontaneous remarks that did not affect the information structure of the child’s responses for the purpose of facilitating the interaction. Prior to the picture-matching game, a picture-naming task was conducted to ensure that the child would use the intended words to refer to the entities in the pictures. This procedure also rendered all the entities in the pictures referentially accessible.

### 2.3 Experimental materials

Sixty question-answer dialogues were embedded in the picture-matching game to elicit 60 SOV sentences with the sentence-medial target object-nouns in five focus conditions, as given below:

- Narrow focus condition: when narrow focus was on the sentence-medial target object-noun, responding to a *what*-question, as illustrated in (1) in the introduction;
- Pre-focus condition: when narrow focus was on the sentence-final verb, responding to a *what-does-X-do-to-Y*-question, as in (4) below;
- Post-focus condition: when narrow focus was on the sentence-initial subject-noun, responding to a *who*-question, as in (5) below;
- Contrastive focus condition: when contrastive focus was on the sentence-medial target object-noun, correcting the experimenter’s remark about the object, as in (2) in the introduction;
- Broad focus condition: when broad focus was over the whole sentence, responding to *what-happens* questions, as in (3) in the introduction.

1. A: Look! The rat, and the bread. It looks like the rat does something to the bread. What does the rat do to the bread?
  B: 쥐가 빵을 [만져요].
  tʃwika p*aŋɨl mantʃʌjo.
  The rat the bread [touches].
2. A: Look! The bread, and someone looks a bit puzzled. It looks like someone looks for the bread. Who looks for the bread?
  B: [소가] 빵을 찾아요.
  soka p*aŋɨl tʃʰatʃajo.
  [The cow] the bread looks for.

Note that narrow focus was included in three sentence-positions to make it possible to examine the effect of narrow focus on the sentence-medial object-nouns compared to the same words in the pre-focus and post-focus conditions, following previous studies on prosodic focus marking (e.g., Chen, 2009). Moreover, comparing the object-nouns in the narrow focus, contrastive focus and broad focus conditions allowed us to examine the prosodic difference between different focus types.

The 60 SOV sentences were unique combinations of five subject-nouns, 12 target object-nouns, and three verbs (Table 1). Each subject-noun was a monosyllabic lexical word followed by a nominative case marker,^6^ /ka/ or /i/. The target object-nouns included six two-syllable (or “short”) words and six four-syllable (or “long”) words, because APs with two to four syllables tend to occur frequently in Korean. The length of the target object-nouns was thus systematically varied to control for any potential effects. Each “short” word was a monosyllabic lexical word followed by an accusative case marker, /ɨl/ or /lɨl/. As for the “long” words, except for /k*amakwilɨl/ (crows), which consisted of a three-syllable lexical word and the accusative case marker /lɨl/, each of the other words consisted of a disyllabic lexical word, a monosyllabic suffix /tɨl/ indicating the plural form of the lexical word, and the accusative case marker /ɨl/. Each target word was initiated with either a high-tone-triggering aspirated stop (i.e., /pʰ/ or /kʰ/) or fortis stop (i.e., /p*/ or /k*/) or a low-tone-triggering lenis stop (i.e., /p/ or /k/) or a vowel (i.e., /a/), so that there would be varied AP tonal patterns in the data. Each target word appeared once in five focus conditions (12 target words × five focus conditions), leading to 60 “sentences” but without subject-nouns and verbs. The five subject-nouns and three verbs were then nearly evenly distributed to the “sentences,” forming 60 SOV sentences. Each sentence was completed with the particle /jo/, a common verb-final politeness marker in informal Korean.

**Table 1:** Words that occurred in the SOV sentences.

Subjects	개가 /kɛka/ “dog”	쥐가 /tʃwika/ “rat”	곰이 /komi/ “bear”	말이 /mali/ “horse”	소가 /soka/ “cow”	
Short objects	발을 /palɨl/ “foot”	비를 /pilɨl/ “rain”	불을 /pulɨl/ “fire”	팔을 /pʰalɨl/ “arm”	빵을 /p*aŋɨl/ “bread”	알을 /alɨl/ “egg”
Long objects	가방들을 /kapaŋtɨlɨl/ “bags”	기둥들을 /kituŋtɨlɨl/ “pillars”	구두들을 /kututɨlɨl/ “shoes”	카드들을 /kʰatɨtɨlɨl/ “cards”	까마귀를 /k*amakwilɨl/ “crow”	안경들을 /ankjʌŋtɨlɨl/ “pairs of glasses”
Verbs	그려 /kɨljʌ/ “draw”	만져 /mantʃʌ/ “touch”	찾아 /tʃʰatʃa/ “look for”			

The 60 sentences were elicited in two experimental sessions: the 30 sentences with “short” target words in Session A and the 30 sentences with “long” target words in Session B. The trials in each session were pseudo-randomized in such a way that trials from the same focus condition did not appear next to each other, and the focused constituent of a trial was not mentioned on its preceding trial.

### 2.4 Experimental procedure

The participants were tested individually upon being given consent by their parents in Hanyang Institute for Phonetics and Cognitive Sciences of Language at Hanyang University. A female native speaker of Seoul Korean administered the experiment after having received intensive training on how to conduct the experiment following the protocol. The experiment lasted about 60 min, including a short chat between the experimenter and the child before the first experimental session, and a short break between the two sessions. Audio recordings were made for each child in each session with a sampling rate of 44.1 kHz with 16-bit resolution. Video recordings were also made for some of the children for training purposes.

### 2.5 Prosodic annotation

The audio recordings from the participants were first orthographically annotated in Praat (Boersma and Weenink, 2013). Then, usable sentences were selected (1,602 or 83% from all the participants in the four age groups; 64% from the 4- to 5-year-olds, 70% from the 7- to 8-year-olds, 80% from the 10- to 11-year-olds, and 91% from the adults), and unusable ones were excluded from further analysis. A target sentence was considered unusable in any of the following cases: (1) the participant produced the target sentence before the experimenter asked the question, (2) the experimenter asked a different question than the intended question on that trial, (3) the experimenter did not provide an adequate description of the picture before she asked a question, (4) the sentence was produced with strong background noise, (5) the sentence was produced with word insertion, deletion or replacement, (6) the sentence was produced with self-repair or clearly perceivable hesitation, or (7) the sentence was produced with perceivable irregular voice quality or intonation caused by cold or unstable emotion.

The usable sentences were then annotated for phrasing, following the Korean Tones and Break Indices (K-ToBI) transcription conventions (Jun, 2000, 2005). That is, the boundaries immediately before the target words (i.e., between the subject-noun and object-noun) and after the target words (i.e., between the object-noun and verb) were annotated as an AP boundary, an IP boundary, or a phrase-internal phonological word boundary (hereafter PW boundary) by combining auditory impression and close inspection of prosodic cues to phrasing (e.g., tonal patterns, boundary tones and breaks). The AP boundary is “a minimal phrasal disjuncture, with no strong subjective sense of pause” and is associated with AP tonal patterns as described in K-ToBI (Jun, 2005, p. 219). Word-final (i.e., pre-boundary) high tone and word-initial (i.e., post-boundary) low tone are taken as the typical AP boundary markers. The absence of voicing in word-initial lenis stops is also an informative indicator of an AP boundary (e.g., Jun, 1993; Cho et al., 2002). Moreover, word-initial (or post-boundary) strengthening in terms of perceptual clarity in the initial segment or syllable may also indicate an AP boundary, unless this cue contradicts another important cue such as a tonal cue. The IP boundary refers to phrasal boundaries that are demarcated by boundary tones and “a strong phrasal disjuncture, with a strong subjective sense of pause,” that is, either an “objective visible pause” or a “virtual pause” cued by final lengthening, as described in K-ToBI (Jun, 2005, p. 219). The PW boundary refers to word boundaries that are not demarcated by perceivable prosodic disjunctures in K-ToBI. It is worth noting that dephrasing does not consistently occur immediately after a focused word in Korean. In such a case, the AP boundary between the focused word and the post-focal word remains, but the pitch span of the post-focal word can be reduced (e.g., Jun and Lee, 1998; Kim et al., 2006; Lee and Xu, 2010). In the present study, when a boundary displayed the above-mentioned features of an AP boundary, but the post-boundary pitch span was noticeably reduced as compared to the pre-boundary pitch span, we annotated this boundary as an AP boundary instead of a PW boundary.

We conducted three rounds of annotation to maximize the reliability and agreement of the annotation. In the first round, the usable sentences were annotated by one transcriber (the first author) without access to information on the experimental conditions, following the above-described K-ToBI conventions, while a portion of the sentences (i.e., 81 sentences produced by two randomly selected participants) were jointly transcribed by two expert K-ToBI transcribers who were native speakers of Korean (the second and third authors), again without access to information on the experimental conditions. The two expert transcribers reached full agreement on the transcription of the 81 sentences. The Cohen’s Kappa test on the annotation of the first transcriber and the expert transcribers for the 81 sentences revealed a very good inter-rater agreement for the boundaries before the target words (*K* = 0.811, *p* < 0.0005), and a good inter-rater agreement after the target words (*K* = 0.644, *p* < 0.0005) (Landis and Koch, 1977). The cases of disagreement were primarily concerned with the distinction between the AP boundary and the PW boundary. In the second round, the first transcriber and the expert transcribers discussed the cases of disagreement, and agreed that the first transcriber should give more weight to three of the cues in her decision on AP and PW boundaries; namely, the word-initial and word-final tones and word-initial strengthening. The first transcriber then re-annotated all the usable sentences without access to the first-round annotation. The expert transcribers then jointly transcribed 23% of the usable sentences (i.e., 10 sentences randomly selected from each participant). The two expert transcribers reached full agreement on the transcription of the 23% sentences. The Cohen’s Kappa test on the second-round annotation for 23% of the usable sentences revealed a very good inter-rater agreement between the first transcriber and the expert transcribers for the boundaries before the target words (*K* = 0.924, *p* < 0.0005), and after the target words (*K* = 0.897, *p* < 0.0005) (Landis and Koch, 1977). To reach a final agreement, a third-round annotation was conducted without access to the previous two rounds of annotation by two additional K-ToBI transcribers, who were native speakers of Korean and did not participate in previous rounds of annotation. The two expert transcribers subsequently examined the ambiguous ones reported by the two additional transcribers, and reached a full agreement on each boundary. The first transcriber then went through the third-round annotation and reached a final agreement with the expert transcribers and the additional transcribers. In this paper, we present an analysis of the data based on the third-round annotation.

## 3 Statistical analyses and results

Having annotated the prosodic boundaries before and after the sentence-medial target words in the sentences, we found that a large proportion of the sentences were produced as three separate APs in all age groups (57.8%^7^ for the 4- to 5-year-olds; 65.7% for the 7- to 8-year-olds; 62.2% for the 10- to 11-year-olds; 46.7% for the adults).

To statistically examine whether and how the children’s use of phrasal boundaries before and after the target words may differ across focus conditions and age groups, we conducted mixed-effects multinomial logistic regression analyses using R Statistical Software (R Core Team, 2015) and the package Brms (Bürkner, 2017, 2018, 2021). Brms adopts a Bayesian approach with the Markov chain Monte Carlo (MCMC) method.

The random factors were speaker (i.e., the participants) and sentence (i.e., the target sentences). The dependent variable was boundary with three categories: AP boundary (reference category), IP boundary, and PW boundary. The independent variables (or fixed effects) were focus and age. focus referred to the focus conditions. For each analysis, we compared narrow focus to another focus condition to address a specific research question, so focus always had two categories. age referred to the four age groups, with the adult group set as the reference category.

Three models were built using the aforementioned factors. Starting from an “empty” model (or Model 0) containing only the random factors, we added the effects of focus and age to form Model 1, following Struiksma et al. (2022). The interaction between focus and age was then added, forming Model 2. The method leave-one-out cross-validation (LOO) was used to evaluate model fit (Vehtari et al., 2017). The model with the lowest estimated looic was regarded as the best-fit model. The boundaries before and after the target words were analyzed separately.

As the model summary of the best-fit model does not straightforwardly show the difference between two focus conditions, or the difference between two focus conditions in each age group in the use of prosodic boundaries, we did follow-up analysis to answer the research questions. When the best-fit model was Model 2, containing the two-way interaction of focus and age, we examined the main effect of focus in each age group, in order to address whether and how the speakers in each age group used prosodic boundaries to distinguish two focus conditions. When the best-fit model was Model 1 containing the main effects of focus and age, we built and summarized a variant of model 1 containing only focus as the fixed factor, in order to address how the speakers varied prosodic boundaries to distinguish two focus conditions, regardless of age. For concision, we report the co-efficient (B) and odds ratio (Exp (B)) from the models in the text; for transparency, we report summaries of these models and the best-fit models in Supplementary Tables 1–12.

### 3.1 Narrow focus vs. pre-focus

**Figure 2:**
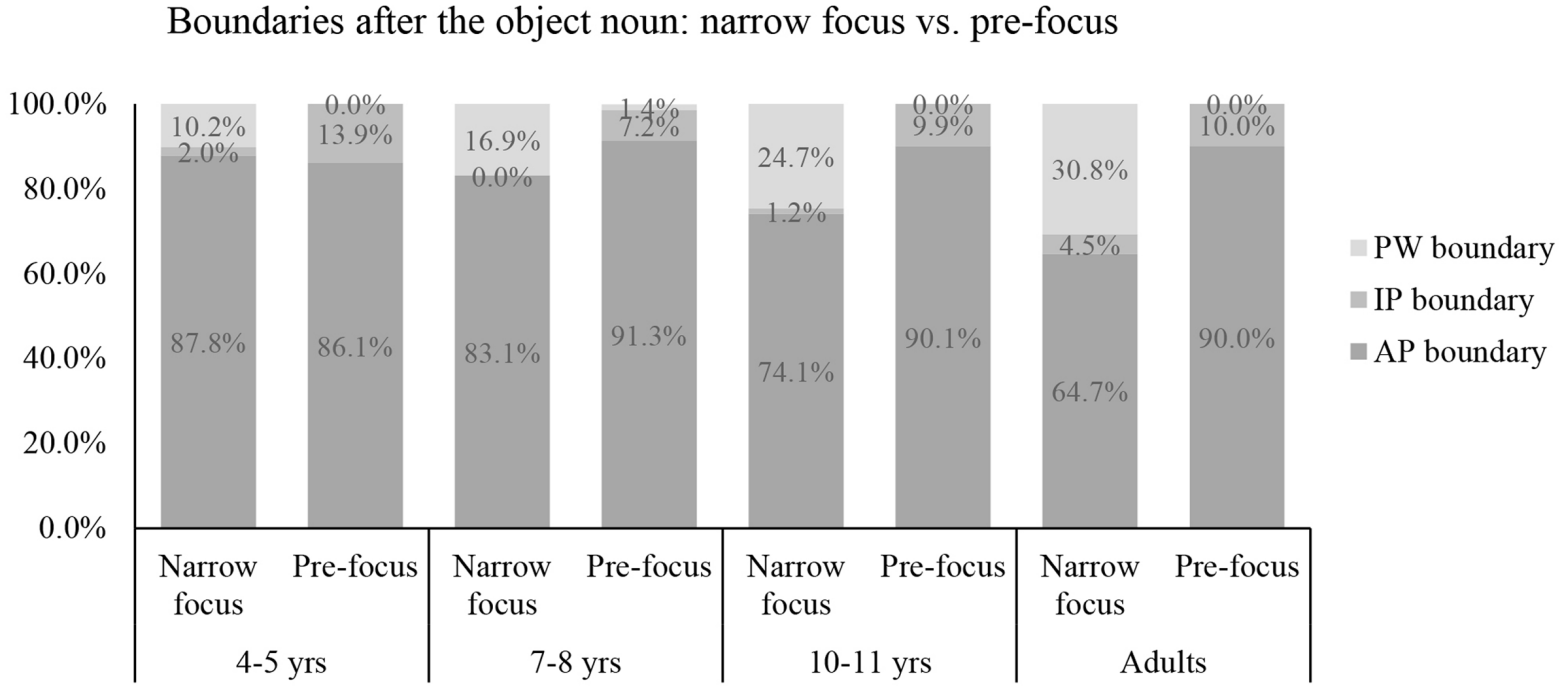
The use of boundaries after the object-noun in the narrow focus condition and pre-focus condition.

Analyzing the boundaries before the target words in the narrow focus and pre-focus conditions (Figure 2), we found that the best-fit model was Model 1 containing the effects of focus and age (looic = 861.1) (Supplementary Table 1). focus thus had a similar effect on the use of boundaries before the target words across age groups. A summary of the model containing only focus (Supplementary Table 2) showed that the odds of the boundary before the target word being an IP boundary rather than an AP boundary in the narrow focus condition were 3.13 times as high as in the pre-focus condition (*B* = −1.13, Exp (*B*) = 0.32). The odds of the boundary before the target word being an PW boundary rather than an AP boundary in the pre-focus condition were 2.53 times as high as in the narrow focus condition (*B* = 0.93, Exp (*B*) = 2.53). In other words, the speakers were more likely to use an IP boundary, but less likely to use a PW boundary before the target word in the narrow focus condition than in the pre-focus condition, regardless of age.

Analyzing the boundaries after the target words, we found that the best-fit model was Model 2, containing the interaction of focus and age (looic = 578.3) (Supplementary Table 3). Subsequent analysis on each age group showed that the model containing focus was the best-fit model for each age group (looic = 242.3 for the adults; looic = 84.3 for the 4- to 5-year-olds; looic = 111.1 for the 7- to 8-year-olds; looic = 143.0 for the 10- to 11-year-olds) (Supplementary Tables 4–7).

For the adults, the odds of the boundary after the target word being an IP boundary rather than an AP boundary in the pre-focus condition were 1.89 times as high as in the narrow focus condition (*B* = 0.64, Exp (*B*) = 1.89). The odds of the boundary after the target word being an PW boundary rather than an AP boundary in the narrow focus condition were 110559.84 times as high as in the pre-focus condition (*B* = −11.61, Exp (*B*) = 0.00). In other words, the adults were less likely to use an IP boundary, but more likely to use a PW boundary after the target word in the narrow focus condition than in the pre-focus condition.

For the 4- to 5-year-olds, the odds of the boundary after the target word being an IP boundary rather than an AP boundary in the pre-focus condition were 10.30 times as high as in the narrow focus condition (*B* = 2.33, Exp (*B*) = 10.30). The odds of the boundary after the target word being an PW boundary rather than an AP boundary in the narrow focus condition were 7131.28 times as high as in the pre-focus condition (*B* = −8.87, Exp (*B*) = 0.00). In other words, the 4- to 5-year-olds were less likely to use an IP boundary, but more likely to use a PW boundary after the target word in the narrow focus condition than in the pre-focus condition.

For the 7- to 8-year-olds, the odds of the boundary after the target word being an IP boundary rather than an AP boundary in the pre-focus condition were 28984.8 times as high as in the narrow focus condition (B = 10.27, Exp (*B*) = 28984.8). The odds of the boundary after the target word being an PW boundary rather than an AP boundary in the narrow focus condition were 33.33 times as high as in the pre-focus condition (*B* = −3.50, Exp (*B*) = 0.03). In other words, the 7- to 8-year-olds were less likely to use an IP boundary, but more likely to use a PW boundary after the target word in the narrow focus condition than in the pre-focus condition.

For the 10- to 11-year-olds, the odds of the boundary after the target word being an IP boundary rather than an AP boundary in the pre-focus condition were 9.79 times as high as in the narrow focus condition (*B* = 2.28, Exp (*B*) = 9.79). The odds of the boundary after the target word being an PW boundary rather than an AP boundary in the narrow focus condition were 64533.95 times as high as in the pre-focus condition (*B* = −11.07, Exp (*B*) = 0.00). In other words, the 10- to 11-year-olds were less likely to use an IP boundary, but more likely to use a PW boundary after the target word in the narrow focus condition than in the pre-focus condition.

As an interim summary, the children used IP boundaries more frequently but used PW boundaries less frequently before the target words in the narrow focus condition than in the pre-focus condition, similar to the adults. These results indicated that the children preferred inserting a large prosodic boundary (IP) immediately before the word in the narrow focus condition, similar to the adults; they also preferred deleting the boundary between the two pre-focal words and producing them as one larger AP or IP when the sentence-final verb was focused, similar to the adults.

As for the boundaries after the target words, the speakers in all age groups used IP boundaries less frequently but used PW boundaries more frequently in the narrow focus condition than in the pre-focus condition. The results indicated that they frequently dephrased the post-focal word in the narrow focus condition, and frequently inserted a large prosodic boundary (IP) immediately before the focused sentence-final word in the pre-focus condition. However, the children differed from the adults in absolute frequency in their use of prosodic boundaries after the target words. The 7- to 8-year-olds were least similar to the adults. To distinguish the narrow focus and pre-focus conditions, while the other age groups seemed to rely more on the use of PW boundaries (or post-focus dephrasing) rather than the use of IP boundaries after the target words, the 7- to 8-year-olds relied more on the use of IP boundaries than the use of PW boundaries after the target words. The 10- to 11-year-olds were most similar to the adults in their use of prosodic boundaries in terms of absolute frequency.

### 3.2 Narrow focus vs. post-focus

Analyzing the boundaries before the target words in the narrow focus and post-focus conditions, we found that the best-fit model was Model 0 containing only the random effects (looic = 863.2), indicating that the speakers did not vary the boundaries before the target words to distinguish narrow focus from post-focus, regardless of age.

Analyzing the boundaries after the target words, we found that the best-fit model was Model 0 containing only the random effects (looic = 659.7), indicating that the speakers did not vary the boundaries after the target words to distinguish narrow focus from post-focus, regardless of age.

As an interim summary, the children did not vary the boundaries before or after the target words to distinguish narrow focus from post-focus, similar to the adults. As the speakers tended to insert an AP or IP boundary before the sentence-medial target word and dephrase its following word when the target word was under narrow focus, this part of the results indicated that they also did so when the target word was post-focus.

### 3.3 Narrow focus vs. broad focus

**Figure 3:**
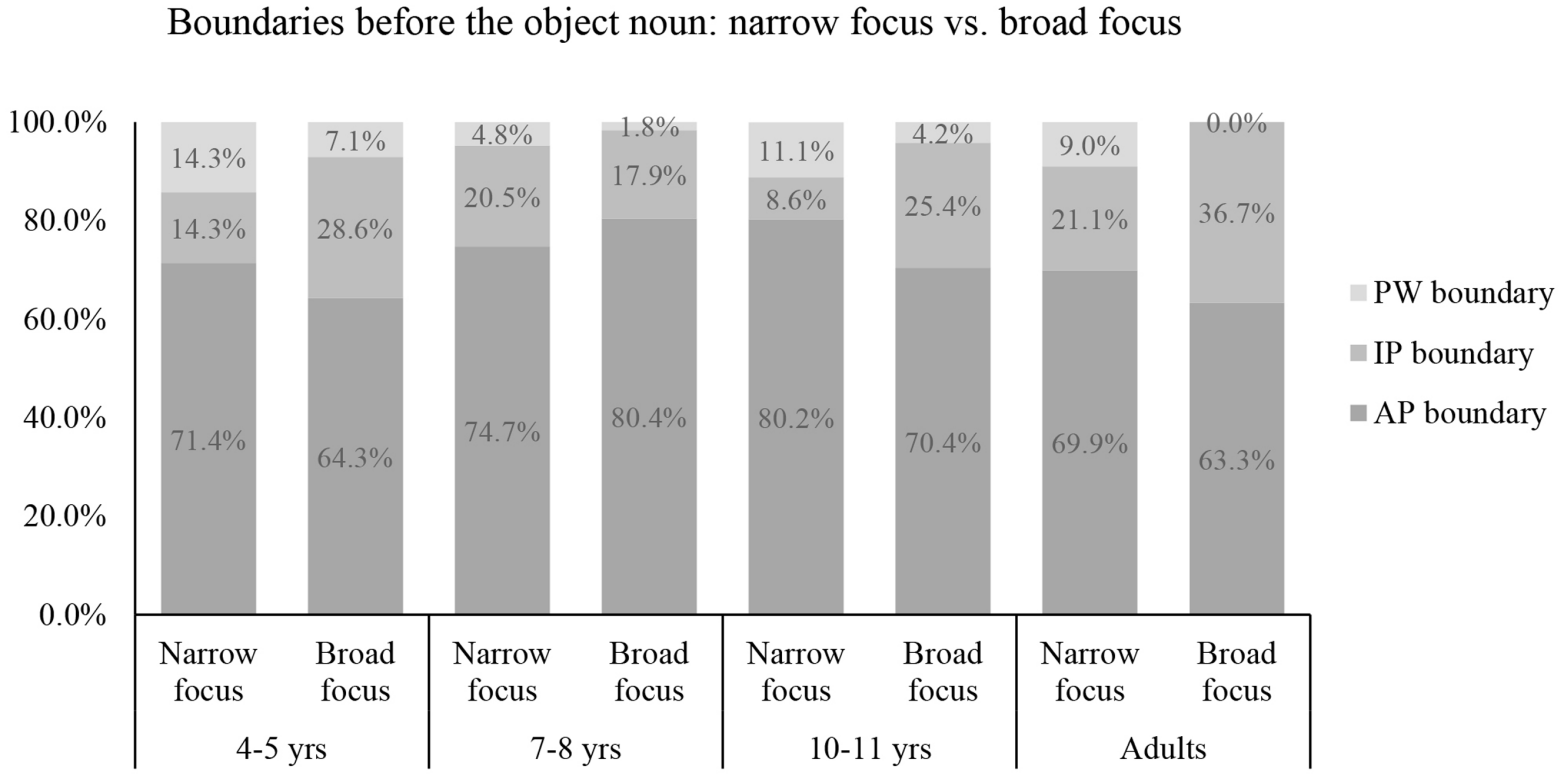
The use of boundaries before the object-noun in the narrow focus condition and broad focus condition.

Analyzing the boundaries before the target words in the narrow focus and broad focus conditions (Figure 3), we found that the best-fit model was Model 2, containing the interaction of focus and age (looic = 782.3) (Supplementary Table 8). Subsequent analysis on each age group showed that the model containing focus was the best-fit model for the 10- to 11-year-olds (looic = 205.8) and the adults (looic = 258.6) (Supplementary Tables 9–10), but the model containing only the random effects was the best-fit model for the 4- to 5-year-olds (looic = 154.6) and 7- to 8-year-olds (looic = 165.9). In other words, the 10- to 11-year-olds varied prosodic boundaries before the target words to distinguish narrow focus from broad focus, similar to the adults, but the 4- to 5-year-olds and 7- to 8-year-olds did not.

For the adults, the odds of the boundary before the target word being an IP boundary rather than an AP boundary in the broad focus condition were 4.08 times as high as in the narrow focus condition (*B* = 1.41, Exp (*B*) = 4.08). The odds of the boundary before the target word being a PW boundary rather than an AP boundary in the narrow focus condition were 26041.80 times as high as in the broad focus condition (*B* = −10.17, Exp (*B*) = 0.00). In other words, the adults were more likely to use an IP boundary, but less likely to use a PW boundary before the target word in the broad focus condition than in the narrow focus condition.

For the 10- to 11-year-olds, the odds of the boundary before the target word being an IP boundary rather than an AP boundary in the broad focus condition were 4.31 times as high as in the narrow focus condition (*B* = 1.46, Exp (*B*) = 4.31). The odds of the boundary before the target word being an PW boundary rather than an AP boundary in the narrow focus condition were 3.13 times as high as in the broad condition (*B* = −1.15, Exp (*B*) = 0.32). In other words, the 10- to 11-year-olds were more likely to use an IP boundary, but less likely to use a PW boundary before the target word in the broad focus condition than in the narrow focus condition, similar to the adults.

Analyzing the boundaries after the target words, we found that the best-fit model was Model 1 containing the effects of focus and age (looic = 670.1) (Supplementary Table 11). focus thus had a similar effect on the use of boundaries after the target words across age groups. A summary of the model containing only focus (Supplementary Table 12) showed that the odds of the boundary after the target word being an IP boundary rather than an AP boundary in the broad focus condition were 5.58 times as high as in the narrow focus condition (*B* = 1.72, Exp (*B*) = 5.58). The odds of the boundary after the target word being an PW boundary rather than an AP boundary in the narrow focus condition were 5.56 times as high as in the broad focus condition (*B* = −1.70, Exp (*B*) = 0.18). In other words, the three groups of children used IP boundaries more frequently but used PW boundaries less frequently after the focal words in the broad focus condition than in the narrow focus condition, similar to the adults.

As an interim summary, the adults used IP boundaries more frequently before and after the target words in the broad focus condition than in the narrow focus condition, indicating their preference for producing the words as separate IPs when the sentence was in broad focus. The adults used PW boundaries more frequently after the target words in the narrow focus condition than in the broad focus condition, indicating their preference for post-focus dephrasing in the narrow focus condition. The 10- to 11-year-olds were adult-like in distinguishing narrow focus from broad focus. However, the two younger groups of children only varied the boundaries after the target words in the same way as the adults did.

### 3.4 Narrow focus vs. contrastive focus

Analyzing the boundaries before the target words in the narrow focus and contrastive focus conditions, we found that the best-fit model was Model 0 containing only the random effects (looic = 854.6), indicating that the speakers did not vary the boundaries before the target words to distinguish narrow focus from contrastive focus, regardless of age.

Analyzing the boundaries after the target words, we found that the best-fit model was Model 0 containing only the random effects (looic = 716.7), indicating that the speakers did not vary the boundaries after the target words to distinguish narrow focus from contrastive focus, regardless of age.

As an interim summary, the children did not vary the boundaries before or after the target words to distinguish narrow focus from contrastive focus, similar to the adults. In other words, the children and adults marked contrastive focus similarly to narrow focus via prosodic phrasing.

## 4 General discussion

To further the current understanding of the effect of transparency of phonological form-meaning mapping on the acquisition of prosodic focus marking, we have examined how Korean-speaking 4- to 5-year-olds, 7- to 8-year-olds, and 10- to 11-year-olds varied prosodic boundaries to distinguish narrow focus from non-focus (i.e., pre-focus and post-focus) and two other types of focus (i.e., broad focus and contrastive focus) in semi-spontaneous production of SOV sentences, as compared to adults.

Regarding the prosodic realization of narrow focus as compared to non-focus, we first compared narrow focus to pre-focus and found that the children in all age groups preferred inserting a large prosodic boundary (i.e., IP boundary) immediately before the focused target word in the narrow focus condition, similar to the adults, and in line with previous findings on Korean-speaking adults’ read speech (e.g., Jun, 1993; Jun and Kim, 2007; Jeon and Nolan, 2017). They also preferred deleting the boundary between the pre-focal words and producing them as one larger AP or IP when the sentence-final verb was focused, similar to the adults. This observation coincides with an earlier finding that pre-focal words tend to be dephrased in adults’ production of read speech (Oh, 1999). Regarding the use of prosodic boundaries after the target words, the children in all age groups frequently used a PW boundary (i.e., dephrasing the post-focal word) in the narrow focus condition, and frequently inserted a large prosodic boundary (IP) immediately before the focused sentence-final word in the pre-focus condition, similar to the adults. However, while the other age groups seemed to rely more on the use of PW boundaries (or post-focus dephrasing) rather than the use of IP boundaries after the target words, the 7- to 8-year-olds relied more on the use of IP boundaries than the use of PW boundaries after the target words, and thus were least similar to the adults. The 10- to 11-year-olds were most similar to the adults in their use of prosodic boundaries in terms of absolute frequency. Regarding the comparison between narrow focus and post-focus, we found that while the children tended to use an AP or IP boundary before the sentence-medial target word and dephrase its following word when the target word was under narrow focus, they also did so when the target word was post-focus, similar to the adults.

Thus, our prediction that Korean-speaking children will be fully adult-like at the age of 7–8 years or later in more frequently using an AP and/or IP boundary before a word and a PW boundary after it in the narrow focus condition than in the non-focus conditions (i.e., pre-focus or post-focus) is only partly borne out. We have unexpectedly observed a protracted developmental path in Korean-speaking children, as the 7- to 8-year-olds relied more on the use of large prosodic boundaries (i.e., IP boundaries) than PW boundaries (related to post-focus dephrasing) to distinguish the narrow focus and pre-focus conditions, different from the other age groups. We suggest two speculations for this finding. First, as the 7- to 8-year-olds start to take read-aloud practice in primary school, their preference to large prosodic boundaries might be from the influence of read speech. However, as previous studies on adults’ read speech usually analyzed different focus conditions separately, we lack comparable findings on how prosodic boundaries are used to distinguish focus conditions in read speech. Moreover, we did not observe a similar pattern in the 10- to 11-year-olds, who had an equal chance of being influenced by read speech in class. Second, the 7- to 8-year-olds might have been more engaged in the picture-matching game, and thus put in more effort in providing answers to the experimenter, compared to the 10- to 11-year-olds and the adults. We speculate that in an edge-prominence language like Korean, more effort in marking focus might lead to more frequent use of prosodic phrasing related to large prosodic boundaries in distinguishing focus conditions. The game was designed in such a way that it would suit the youngest children in the study. It is thus plausible that the oldest children and the adults did not put in more effort in production than needed in encoding focus. A similar observation about 10- to 11-year-olds engaging less with the game and making less effort to answer questions than younger children was reported in Romøren and Chen (2021).

As for the distinction between narrow focus and broad focus, only the 10- to 11-year-olds were fully adult-like. They preferred producing the words as separate IPs when the sentence was in broad focus, and preferred post-focus dephrasing in the narrow focus condition. However, the two younger groups of children only varied the boundaries after the target words in the same way as the adults did to distinguish the two focus conditions. These results largely support our prediction concerning the effect of focal constituent size.

Regarding the comparison between narrow focus and contrastive focus, we did not find any evidence of the speakers distinguishing the two types of focus using prosodic boundaries, regardless of age. The results fully support our prediction regarding the effect of contrastivity.

In previous studies on the acquisition of phonological focus marking, Dutch-speaking children used accentuation close to the ceiling across narrow focus, broad focus, and contrastive focus conditions, showing no evidence of using accent placement in distinguishing focus types, similar to Dutch-speaking adults (Romøren, 2016), although the exact accent types used for marking the three types of focus were not reported in Romøren (2016). Swedish-speaking children used the prominence-marking high tone more frequently in narrow focus than in broad focus, but did not distinguish narrow focus from contrastive focus using this cue, similar to Swedish-speaking adults (Romøren, 2016). Thus, the current findings, along with those from previous studies, imply that languages differ both in whether and how focus types are distinguished by language-specific phonological cues, and in the acquisition of phonological marking of these focus types.

Based on the findings of the present study, we can depict Korean-speaking children’s developmental path of phonological focus marking from the age of 4 to 11 as follows: At the age of 4 to 5, Korean-speaking children use prosodic phrasing to mark focus and distinguish different types of focus in a largely adult-like manner, though their use of prosodic boundaries for focus-marking purposes is not fully adult-like in terms of absolute frequency. In contrast, 4- to 5-year-old Swedish-speaking children were fully adult-like in the use of the prominence-marking high tone in sentence-final position, and largely adult-like in sentence-medial position in terms of manner and frequency (Romøren, 2016; Romøren and Chen, 2021). The difference between Korean-speaking and Swedish-speaking children is further evident at later stages. For example, at the age of 7 to 8, Korean-speaking children tend to rely more on the use of large prosodic boundaries (i.e., IP boundaries) than PW boundaries (or dephrasing) when distinguishing narrow focus from pre-focus, different from adults. At the age of 10 to 11, they exhibit fully adult-like abilities in distinguishing focus conditions. The results in general support our hypothesis regarding Korean-speaking children’s rate of acquisition of phonological focus marking, compared to that of Swedish-speaking children and Dutch-speaking children.

The results have further implications for understanding cross-linguistic variation in the acquisition of focus marking. As discussed at the outset of the paper, Swedish employs a highly transparent phonological means of focus marking, so that words under narrow focus are consistently assigned a word-final high tone (Bruce, 2007; Romøren, 2016). In other words, Swedish demonstrates a clear and direct mapping between the phonological form and the focus-related meaning. On the other hand, while focus marking in Korean is typically achieved through phrasing (involving the initiation of a large prosodic constituent such as an IP) (e.g., Jun, 1993, 1998, 2000, 2005), phrasing is not exclusively used for focus marking, indicating a less transparent form-meaning mapping compared to that observed in Swedish. Given these cross-linguistic differences between Swedish and Korean, our results lend support to Chen’s (2018) view that the transparency of the form-meaning mapping between phonological cues and focus conditions influences the rate of acquisition in prosodic focus marking across languages.

Let us now compare our Korean results with those observed in Dutch-speaking children. Recall that Dutch-speaking 4- to 5-year-olds were not fully adult-like in their choice of accent type for focus-marking purposes (Chen, 2011b). In contrast, at these ages (4 to 5 years), Korean-speaking children exhibited some phonological focus marking patterns that were more adult-like than their Dutch-speaking peers. Thus, our results suggest that Korean-speaking children tend to acquire an adult-like way of focus marking relatively earlier than their Dutch-speaking peers. However, the difference between Korean-speaking and Dutch-speaking children does not seem to be fully in line with the effect of transparency based on consistency in the association between a form and a meaning. This is because there is no apparent difference in the consistency of the form-meaning mapping between Korean and Dutch. In other words, phrasing and pitch accent, which are used for focus marking in each language, respectively, can also occur in various other non-focused contexts. However, our results are compatible with a transparency hypothesis based on perceptual transparency between a form and a meaning. That is, if we extend the notion of the degree of transparency in the form-meaning mapping to include perceptual transparency of the cues to focus, the earlier acquisition of focus marking by Korean-speaking children can still be understood as a reflection of cross-linguistic differences in the transparency of the form-meaning mapping.

## 5 Conclusion and limitations

In conclusion, our findings on Korean-speaking children support Chen’s (2018) view that a higher degree of transparency in the form-meaning mapping between phonological cues and focus leads to a faster rate of acquisition in prosodic focus marking. Further, we demonstrate that a greater diversity in phonological forms, such as the use of phrasing for purposes other than focus marking in Korean, can slow down the rate of acquisition. Thus, our study not only provides new experimental evidence for the role of transparency in form-meaning mapping as a determinant of children’s acquisition of focus marking, but also expands our current understanding of the notion of the degree of transparency in the form-meaning mapping to include perceptual salience of the phonological cue.

Some important questions remain to be addressed in future studies. For example, we did not observe evidence of Korean-speaking adults and children distinguishing narrow focus from post-focus as well as narrow focus from contrastive focus using prosodic phrasing. Given that these focus conditions are distinguished phonologically and/or phonetically in many other languages, they may be distinguished with pitch- and duration-related phonetic cues as well as segmental cues in Korean. It will be insightful to study whether and how other phonetic focus-marking cues are used in Korean when the primary cue, prosodic phrasing, does not suffice to distinguish two focus conditions (but see Cho et al., 2011; Hatcher et al., 2023, for related data), as well as how Korean-speaking children acquire the use of the phonetic cues. Moreover, as Korean utilizes both the left and right edges of prosodic phrases for phrasing and focus marking, another interesting question for future research is whether our findings on the acquisition of prosodic focus marking in Korean-speaking children can be generalized to children acquiring other edge-prominence languages, like Mongolian, in which prosodic phrasing and focus are typically marked at the left edge of prosodic units (Karlsson, 2014). Thus, more studies under the same theoretical framework adopting similar experimental methods need to be done to broaden our understanding of the acquisition of prosodic focus marking.

## Data availability statement

The raw data supporting the conclusions of this article will be made available by the authors, without undue reservation.

## Ethics statement

Ethical review and approval were not required for the study on human participants in accordance with the local legislation and institutional requirements at the time of testing. Written informed consent for participation in this study was provided by the participants’ legal guardians/next of kin.

## Author contributions

AY: Conceptualization, Data curation, Formal analysis, Funding acquisition, Investigation, Methodology, Resources, Validation, Visualization, Writing – original draft, Writing – review & editing. TC: Conceptualization, Data curation, Funding acquisition, Investigation, Methodology, Resources, Validation, Writing – review & editing. SK: Conceptualization, Data curation, Funding acquisition, Investigation, Methodology, Resources, Validation, Writing – review & editing. AC: Conceptualization, Data curation, Funding acquisition, Investigation, Methodology, Project administration, Resources, Supervision, Validation, Writing – review & editing.

## Footnotes

^1^Focus can carry contrastive information, e.g., a correction to a certain piece of information introduced previously or an alternative to what has been mentioned already. While the focal items are regarded as being in contrastive focus in both conditions, the former is also referred to as corrective focus (Gussenhoven, 2004, 2007). In the present study, we use “contrastive focus” instead of “corrective focus” to refer to the former condition.

^2^The examples are from the experimental materials of the present study. The referents are referred to with the definite article in the English glossary because they had been introduced in a picture-naming task before the production experiment.

^3^The focused constituents are in square brackets in the examples.

^4^An Intermediate Phrase (ip), which is larger than an AP but smaller than an IP, was added in a later version of the Korean prosodic hierarchy (Jun, 2007b, 2014). However, as ip is not clearly defined, transcribing ips in speech data is not straightforward, and its role in focus marking is not examined in the present study.

^5^The present study was done using an adapted method from Chen (2011a,b), parallel to Romøren and Chen (2021). The results may thus be more comparable to Chen (2011a,b) on Dutch and Romøren and Chen (2021) on Swedish than to other previous studies.

^6^There are two nominative case markers (i.e., /ka/ and /i/) and two accusative case markers (/ɨl/ and /lɨl/) in Korean. The choice between each two variants depends on the presence of a coda consonant in the preceding noun.

^7^The percentages provided in the parentheses were calculated based on raw data.
