# Supplementary material for: Prosodic focus marking in Seoul Korean-speaking children: the use of prosodic phrasing
Source: Front Psychol. 2024 Jul 31;15:1352280. doi: 10.3389/fpsyg.2024.1352280 (PMC11331792; doi:10.3389/fpsyg.2024.1352280)
Supplement: Supplementary file 1 [file Data_Sheet_1.PDF]

Supplementary Material

Prosodic focus marking in Seoul Korean-speaking children: The use of prosodic phrasing

Anqi Yang*, Taehong Cho, Sahyang Kim, Aoju Chen*

*** Correspondence:** Anqi Yang: anqi.yang@tju.edu.cn, Aoju Chen: aoju.chen@uu.nl

1 Supplementary Tables

Supplementary Table 1: The best-fit model (Model 1) for the analysis of the boundaries before the target words, comparing narrow focus to pre-focus.

Group-Level Effects:							
~sentence (Number of levels: 24)							
	Estimate	Est.Error	l-95% CI	u-95% CI	Rhat	Bulk_ESS	Tail_ESS
sd(muIP_Intercept)	0.39	0.24	0.02	0.88	1.00	2403	3418
sd(muPW_Intercept)	0.63	0.22	0.21	1.10	1.00	2654	2616
~speaker (Number of levels: 34)							
	Estimate	Est.Error	l-95% CI	u-95% CI	Rhat	Bulk_ESS	Tail_ESS
sd(muIP_Intercept)	1.46	0.34	0.92	2.24	1.00	2865	4509
sd(muPW_Intercept)	1.29	0.27	0.84	1.90	1.00	3101	5033
Population-Level Effects:							
	Estimate	Est.Error	l-95% CI	u-95% CI	Rhat	Bulk_ESS	Tail_ESS
muIP_Intercept	-2.14	0.57	-3.34	-1.10	1.00	3549	4025
muPW_Intercept	-2.58	0.52	-3.64	-1.59	1.00	4358	4901
muIP_age_10_11	-0.29	0.84	-1.91	1.41	1.00	4262	4810
muIP_age_4_5	0.43	0.90	-1.31	2.32	1.00	3854	4082
muIP_age_7_8	0.49	0.81	-1.10	2.12	1.00	3566	4748
muIP_prefocus	-1.14	0.36	-1.87	-0.46	1.00	7864	5096
muPW_age_10_11	-0.06	0.70	-1.47	1.32	1.00	3998	5267
muPW_age_4_5	0.20	0.80	-1.34	1.77	1.00	4660	5165
muPW_age_7_8	-0.58	0.72	-2.04	0.79	1.00	4377	5210
muPW_prefocus	0.95	0.39	0.16	1.73	1.00	5849	5281
Exp(B):							
	Estimate	Est.Error	Q2.5	Q97.5			
muIP_Intercept	0.12	1.77	0.04	0.33			
muPW_Intercept	0.08	1.68	0.03	0.20			
muIP_age_10_11	0.75	2.31	0.15	4.10			
muIP_age_4_5	1.54	2.47	0.27	10.20			
muIP_age_7_8	1.63	2.25	0.33	8.33			
muIP_prefocus	0.32	1.43	0.15	0.63			
muPW_age_10_11	0.94	2.02	0.23	3.75			
muPW_age_4_5	1.23	2.23	0.26	5.85			
muPW_age_7_8	0.56	2.06	0.13	2.21			
muPW_prefocus	2.58	1.48	1.18	5.64			

Supplementary Table 2: The model containing only FOCUS for the analysis of the boundaries before the target words, comparing narrow focus to pre-focus.

Group-Level Effects:							
~sentence (Number of levels: 24)							
	Estimate	Est.Error	l-95% CI	u-95% CI	Rhat	Bulk_ESS	Tail_ESS
sd(muIP_Intercept)	0.39	0.23	0.03	0.89	1.00	1263	1641
sd(muPW_Intercept)	0.61	0.22	0.17	1.08	1.00	1114	849
~speaker (Number of levels: 34)							
	Estimate	Est.Error	l-95% CI	u-95% CI	Rhat	Bulk_ESS	Tail_ESS
sd(muIP_Intercept)	1.35	0.30	0.86	1.99	1.00	1603	2149
sd(muPW_Intercept)	1.22	0.25	0.80	1.77	1.01	1452	2062
Population-Level Effects:							
	Estimate	Est.Error	l-95% CI	u-95% CI	Rhat	Bulk_ESS	Tail_ESS
muIP_Intercept	-1.97	0.34	-2.68	-1.35	1.00	1764	2400
muPW_Intercept	-2.63	0.37	-3.38	-1.95	1.00	2047	2595
muIP_prefocus	-1.13	0.35	-1.87	-0.47	1.00	2763	1904
muPW_prefocus	0.93	0.38	0.20	1.69	1.00	2726	2515
Exp(B):							
	Estimate	Est.Error	Q2.5	Q97.5			
muIP_Intercept	0.14	1.40	0.07	0.26			
muPW_Intercept	0.07	1.44	0.03	0.14			
muIP_prefocus	0.32	1.42	0.15	0.63			
muPW_prefocus	2.53	1.46	1.23	5.41			

Supplementary Table 3: The best-fit model (Model 2) for the analysis of the boundaries after the target words, comparing narrow focus to pre-focus.

Group-Level Effects:							
~sentence (Number of levels: 24)							
	Estimate	Est.Error	l-95% CI	u-95% CI	Rhat	Bulk_ESS	Tail_ESS
sd(muIP_Intercept)	0.77	0.33	0.18	1.47	1.01	1233	1233
sd(muPW_Intercept)	0.62	0.30	0.10	1.29	1.00	915	920
~speaker (Number of levels: 34)							
	Estimate	Est.Error	l-95% CI	u-95% CI	Rhat	Bulk_ESS	Tail_ESS
sd(muIP_Intercept)	1.58	0.52	0.78	2.78	1.00	881	1583
sd(muPW_Intercept)	1.80	0.40	1.12	2.69	1.00	1064	1518
Population-Level Effects:							
	Estimate	Est.Error	l-95% CI	u-95% CI	Rhat	Bulk_ESS	Tail_ESS
muIP_Intercept	-3.84	0.85	-5.70	-2.37	1.00	1585	2352
muPW_Intercept	-1.23	0.65	-2.55	0.01	1.01	1019	1911
muIP_age_10_11	-1.57	1.58	-5.07	1.26	1.00	1209	1607
muIP_age_4_5	-1.20	1.66	-4.86	1.73	1.00	1272	1541
muIP_age_7_8	-35.58	75.52	-147.36	-2.03	1.02	194	55
muIP_prefocus	0.74	0.68	-0.58	2.14	1.00	1995	2502
muIP_age_10_11:prefocus	1.62	1.44	-0.82	4.71	1.00	1565	1676
muIP_age_4_5:prefocus	1.85	1.53	-0.73	5.27	1.00	1770	1622
muIP_age_7_8:prefocus	35.28	75.50	1.70	146.30	1.02	190	55
muPW_age_10_11	-0.46	0.97	-2.48	1.34	1.01	1009	1513
muPW_age_4_5	-1.59	1.12	-3.81	0.59	1.00	1195	2086
muPW_age_7_8	-1.12	0.99	-3.11	0.72	1.00	928	1827
muPW_prefocus	-298.02	593.10	-2100.90	-6.11	1.34	9	13
muPW_age_10_11:prefocus	-45.47	465.16	-1591.82	842.74	1.18	76	34
muPW_age_4_5:prefocus	-742.66	2263.10	-9527.62	535.32	1.22	46	12
muPW_age_7_8:prefocus	294.73	593.06	2.56	2098.75	1.34	9	13
Exp (B):							
	Estimate	Est.Error	Q2.5	Q97.5			
muIP_Intercept	0.02	2.33	0.00	0.09			
muPW_Intercept	0.29	1.91	0.08	1.01			
muIP_age_10_11	0.21	4.84	0.01	3.52			
muIP_age_4_5	0.30	5.27	0.01	5.65			
muIP_age_7_8	0.00	6.28e+32	0.00	0.13			
muIP_prefocus	2.09	1.97	0.56	8.50			
muIP_age_10_11:prefocus	5.05	4.23	0.44	110.53			
muIP_age_4_5:prefocus	6.39	4.63	0.48	194.14			
muIP_age_7_8:prefocus	2.10e+15	6.16e+32	5.46	3.44e+63			
muPW_age_10_11	0.63	2.63	0.08	3.82			
muPW_age_4_5	0.20	3.08	0.02	1.80			
muPW_age_7_8	0.33	2.68	0.04	2.06			
muPW_prefocus	0.00	3.80e+257	0.00	0.00			
muPW_age_10_11:prefocus	0.00	1.03e+202	0.00	Inf			
muPW_age_4_5:prefocus	0.00	Inf	0.00	3.06e+232			
muPW_age_7_8:prefocus	1.00e+128	3.66e+257	12.93	Inf			

Supplementary Table 4: The main effect of FOCUS model for the analysis of the boundaries after the target words, comparing narrow focus to pre-focus, for the adults.

	Estimate	Est.Error	l-95% CI	u-95% CI	Rhat	Bulk_ESS	Tail_ESS
Group-Level Effects:							
~sentence (Number of levels: 24)							
	Estimate	Est.Error	l-95% CI	u-95% CI	Rhat	Bulk_ESS	Tail_ESS
sd(muIP_Intercept)	1.31	0.61	0.26	2.66	1.00	2070	1906
sd(muPW_Intercept)	0.65	0.41	0.04	1.58	1.00	2561	3254
~speaker (Number of levels: 12)							
	Estimate	Est.Error	l-95% CI	u-95% CI	Rhat	Bulk_ESS	Tail_ESS
sd(muIP_Intercept)	1.96	0.89	0.70	4.17	1.00	3107	5076
sd(muPW_Intercept)	2.19	0.74	1.12	3.96	1.00	2925	4901
Population-Level Effects:							
	Estimate	Est.Error	l-95% CI	u-95% CI	Rhat	Bulk_ESS	Tail_ESS
muIP_Intercept	-4.12	1.05	-6.43	-2.31	1.00	4233	4671
muPW_Intercept	-1.11	0.77	-2.73	0.42	1.00	3798	5426
muIP_prefocus	0.64	0.89	-1.14	2.34	1.00	5414	4908
muPW_prefocus	-11.61	8.27	-33.64	-4.47	1.00	2337	1314
Exp(B):							
	Estimate	Est.Error	Q2.5	Q97.5			
muIP_Intercept	0.02	2.86	0.00	0.10			
muPW_Intercept	0.33	2.17	0.07	1.52			
muIP_prefocus	1.89	2.43	0.32	10.35			
muPW_prefocus	0.00	3895.01	0.00	0.01			

Supplementary Table 5: The main effect of FOCUS model for the analysis of the boundaries after the target words, comparing narrow focus to pre-focus, for the four- to five-year-olds.

Group-Level Effects:							
~sentence (Number of levels: 24)							
	Estimate	Est.Error	l-95% CI	u-95% CI	Rhat	Bulk_ESS	Tail_ESS
sd(muIP_Intercept)	0.71	0.60	0.02	2.25	1.00	3601	4398
sd(muPW_Intercept)	1.15	0.91	0.04	3.45	1.00	3414	3396
~speaker (Number of levels: 6)							
	Estimate	Est.Error	l-95% CI	u-95% CI	Rhat	Bulk_ESS	Tail_ESS
sd(muIP_Intercept)	1.14	1.00	0.04	3.65	1.00	2804	4327
sd(muPW_Intercept)	1.15	1.03	0.04	3.78	1.00	3548	3985
Population-Level Effects:							
	Estimate	Est.Error	l-95% CI	u-95% CI	Rhat	Bulk_ESS	Tail_ESS
muIP_Intercept	-4.42	1.40	-7.68	-2.19	1.00	5762	3841
muPW_Intercept	-2.69	1.10	-5.23	-0.91	1.00	7011	6552
muIP_prefocus	2.33	1.37	0.03	5.40	1.00	7270	4141
muPW_prefocus	-8.87	9.05	-36.21	-0.68	1.00	2520	1515
Exp(B):							
	Estimate	Est.Error	Q2.5	Q97.5			
muIP_Intercept	0.01	4.05	0.00	0.11			
muPW_Intercept	0.07	2.99	0.01	0.40			
muIP_prefocus	10.30	3.92	1.03	222.37			
muPW_prefocus	0.00	8515.50	0.00	0.51			

Supplementary Table 6: The main effect of FOCUS model for the analysis of the boundaries after the target words, comparing narrow focus to pre-focus, for the seven- to eight-year-olds.

Group-Level Effects:							
~sentence (Number of levels: 24)							
	Estimate	Est.Error	l-95% CI	u-95% CI	Rhat	Bulk_ESS	Tail_ESS
sd(muIP_Intercept)	1.84	1.41	0.10	5.29	1.00	2843	3696
sd(muPW_Intercept)	1.51	0.81	0.21	3.36	1.00	1920	1999
~speaker (Number of levels: 8)							
	Estimate	Est.Error	l-95% CI	u-95% CI	Rhat	Bulk_ESS	Tail_ESS
sd(muIP_Intercept)	2.72	2.02	0.24	7.89	1.00	2442	2391
sd(muPW_Intercept)	2.14	1.10	0.63	4.81	1.00	2938	3211
Population-Level Effects:							
	Estimate	Est.Error	l-95% CI	u-95% CI	Rhat	Bulk_ESS	Tail_ESS
muIP_Intercept	-14.60	10.98	-45.63	-4.64	1.00	2376	1427
muPW_Intercept	-2.53	1.13	-5.10	-0.52	1.00	4129	4634
muIP_prefocus	10.27	10.39	1.30	40.04	1.00	2436	1422
muPW_prefocus	-3.50	1.64	-7.32	-0.91	1.00	4839	3540
Exp(B)							
	Estimate	Est.Error	Q2.5	Q97.5			
muIP_Intercept	0.00	58523.76	0.00	0.01			
muPW_Intercept	0.08	3.10	0.01	0.59			
muIP_prefocus	28984.80	32518.82	3.65	2.44e+17			
muPW_prefocus	0.03	5.16	0.00	0.40			

Supplementary Table 7: The main effect of FOCUS model for the analysis of the boundaries after the target words, comparing narrow focus to pre-focus, for the ten- to eleven-year-olds.

Group-Level Effects:							
~sentence (Number of levels: 24)							
	Estimate	Est.Error	l-95% CI	u-95% CI	Rhat	Bulk_ESS	Tail_ESS
sd(muIP_Intercept)	0.89	0.70	0.04	2.63	1.00	2788	3861
sd(muPW_Intercept)	0.63	0.47	0.03	1.75	1.00	3520	4145
~speaker (Number of levels: 8)							
	Estimate	Est.Error	l-95% CI	u-95% CI	Rhat	Bulk_ESS	Tail_ESS
sd(muIP_Intercept)	1.59	1.14	0.13	4.43	1.00	2600	3545
sd(muPW_Intercept)	1.94	0.90	0.73	4.16	1.00	3197	5141
Population-Level Effects:							
	Estimate	Est.Error	l-95% CI	u-95% CI	Rhat	Bulk_ESS	Tail_ESS
muIP_Intercept	-5.18	1.54	-8.71	-2.76	1.00	4856	3595
muPW_Intercept	-1.45	0.87	-3.26	0.26	1.00	4581	4998
muIP_prefocus	2.28	1.36	-0.01	5.36	1.00	7425	4299
muPW_prefocus	-11.07	10.37	-41.27	-2.99	1.00	1311	580
Exp(B):							
	Estimate	Est.Error	Q2.5	Q97.5			
muIP_Intercept	0.01	4.66	0.00	0.06			
muPW_Intercept	0.23	2.40	0.04	1.29			
muIP_prefocus	9.79	3.89	0.99	212.01			
muPW_prefocus	0.00	32021.35	0.00	0.05			

Supplementary Table 8: The best-fit model (Model 2) for the analysis of the boundaries before the target words, comparing narrow focus to broad focus.

	Estimate	Est.Error	l-95% CI	u-95% CI	Rhat	Bulk_ESS	Tail_ESS
Group-Level Effects:							
~sentence (Number of levels: 24)							
	Estimate	Est.Error	l-95% CI	u-95% CI	Rhat	Bulk_ESS	Tail_ESS
sd(muIP_Intercept)	0.27	0.18	0.01	0.66	1.00	1019	1101
sd(muPW_Intercept)	0.48	0.33	0.02	1.26	1.00	1102	1507
~speaker (Number of levels: 34)							
	Estimate	Est.Error	l-95% CI	u-95% CI	Rhat	Bulk_ESS	Tail_ESS
sd(muIP_Intercept)	1.85	0.36	1.28	2.66	1.00	1313	1984
sd(muPW_Intercept)	1.55	0.44	0.87	2.58	1.00	1587	2371
Population-Level Effects:							
	Estimate	Est.Error	l-95% CI	u-95% CI	Rhat	Bulk_ESS	Tail_ESS
muIP_Intercept	-2.33	0.69	-3.77	-1.07	1.01	919	1586
muPW_Intercept	-3.06	0.74	-4.69	-1.78	1.00	1615	1758
muIP_age_10_11	-0.49	1.03	-2.47	1.56	1.00	1081	1824
muIP_age_4_5	0.35	1.13	-1.73	2.68	1.01	1067	1810
muIP_age_7_8	0.42	1.00	-1.45	2.45	1.01	817	1781
muIP_broadfocus	1.23	0.40	0.48	2.03	1.00	2278	2587
muIP_age_10_11:broadfocus	0.22	0.65	-1.04	1.54	1.00	3195	2825
muIP_age_4_5:broadfocus	-0.38	0.70	-1.74	0.98	1.00	3095	3465
muIP_age_7_8:broadfocus	-1.48	0.62	-2.69	-0.34	1.00	3127	3339
muPW_age_10_11	0.40	0.99	-1.59	2.39	1.00	1544	2128
muPW_age_4_5	0.75	1.08	-1.40	2.92	1.00	1418	1921
muPW_age_7_8	-0.41	1.06	-2.49	1.73	1.00	1931	2182
muPW_broadfocus	-18.20	17.56	-73.61	-2.93	1.01	508	284
muPW_age_10_11:broadfocus	17.15	17.57	1.63	72.48	1.01	507	287
muPW_age_4_5:broadfocus	17.44	17.58	1.99	73.08	1.01	521	289
muPW_age_7_8:broadfocus	16.72	17.61	0.72	72.00	1.01	498	295
Exp(B):							
	Estimate	Est.Error	Q2.5	Q97.5			
muIP_Intercept	0.10	1.99	0.02	0.34			
muPW_Intercept	0.05	2.09	0.01	0.17			
muIP_age_10_11	0.61	2.80	0.08	4.75			
muIP_age_4_5	1.42	3.10	0.18	14.53			
muIP_age_7_8	1.52	2.71	0.23	11.57			
muIP_broadfocus	3.44	1.49	1.61	7.64			
muIP_age_10_11:broadfocus	1.24	1.91	0.35	4.66			
muIP_age_4_5:broadfocus	0.69	2.01	0.18	2.65			
muIP_age_7_8:broadfocus	0.23	1.86	0.07	0.72			
muPW_age_10_11	1.49	2.70	0.20	10.89			
muPW_age_4_5	2.12	2.93	0.25	18.51			
muPW_age_7_8	0.67	2.87	0.08	5.62			
muPW_broadfocus	0.00	4.25e+07	0.00	0.05			
muPW_age_10_11:broadfocus	2.80e+07	4.27e+07	5.11	2.99e+31			
muPW_age_4_5:broadfocus	3.75e+07	4.29e+07	7.33	5.45e+31			
muPW_age_7_8:broadfocus	1.82e+07	4.44e+07	2.05	1.85e+31			

Supplementary Table 9: The main effect of FOCUS model for the analysis of the boundaries before the target words, comparing narrow focus to broad focus, for the adults.

	Estimate	Est.Error	l-95% CI	u-95% CI	Rhat	Bulk_ESS	Tail_ESS
Group-Level Effects:							
~sentence (Number of levels: 24)							
	Estimate	Est.Error	l-95% CI	u-95% CI	Rhat	Bulk_ESS	Tail_ESS
sd(muIP_Intercept)	0.56	0.34	0.03	1.32	1.00	2391	3481
sd(muPW_Intercept)	0.67	0.51	0.03	1.90	1.00	3511	3873
~speaker (Number of levels: 12)							
	Estimate	Est.Error	l-95% CI	u-95% CI	Rhat	Bulk_ESS	Tail_ESS
sd(muIP_Intercept)	3.13	1.03	1.71	5.52	1.00	3151	4041
sd(muPW_Intercept)	2.12	0.95	0.80	4.40	1.00	3432	3675
Population-Level Effects:							
	Estimate	Est.Error	l-95% CI	u-95% CI	Rhat	Bulk_ESS	Tail_ESS
muIP_Intercept	-2.61	1.00	-4.72	-0.74	1.00	2511	3161
muPW_Intercept	-3.33	1.05	-5.73	-1.68	1.00	6059	6624
muIP_broadfocus	1.41	0.50	0.50	2.41	1.00	8711	4686
muPW_broadfocus	-10.17	8.32	-33.77	-2.39	1.00	2321	1357
Exp(B):							
	Estimate	Est.Error	Q2.5	Q97.5			
muIP_Intercept	0.07	2.73	0.01	0.48			
muPW_Intercept	0.04	2.85	0.00	0.19			
muIP_broadfocus	4.08	1.64	1.65	11.19			
muPW_broadfocus	0.00	4111.03	0.00	0.09			

Supplementary Table 10: The main effect of FOCUS model for the analysis of the boundaries before the target words, comparing narrow focus to broad focus, for the ten- to eleven-year-olds.

Group-Level Effects:							
~sentence (Number of levels: 24)							
	Estimate	Est.Error	l-95% CI	u-95% CI	Rhat	Bulk_ESS	Tail_ESS
sd(muIP_Intercept)	0.64	0.46	0.03	1.75	1.00	2167	3735
sd(muPW_Intercept)	0.84	0.63	0.03	2.40	1.00	2494	2997
~speaker (Number of levels: 8)							
	Estimate	Est.Error	l-95% CI	u-95% CI	Rhat	Bulk_ESS	Tail_ESS
sd(muIP_Intercept)	1.30	0.65	0.33	2.89	1.00	2878	2841
sd(muPW_Intercept)	1.71	0.97	0.44	4.05	1.00	3221	4099
Population-Level Effects:							
	Estimate	Est.Error	l-95% CI	u-95% CI	Rhat	Bulk_ESS	Tail_ESS
muIP_Intercept	-2.69	0.74	-4.29	-1.37	1.00	5236	4558
muPW_Intercept	-2.62	0.91	-4.67	-1.06	1.00	5571	4708
muIP_broadfocus	1.46	0.65	0.27	2.88	1.00	8816	5186
muPW_broadfocus	-1.15	1.00	-3.37	0.54	1.00	6388	2663
Exp(B):							
	Estimate	Est.Error	Q2.5	Q97.5			
muIP_Intercept	0.07	2.10	0.01	0.25			
muPW_Intercept	0.07	2.47	0.01	0.35			
muIP_broadfocus	4.32	1.91	1.31	17.87			
muPW_broadfocus	0.32	2.73	0.03	1.72			

Supplementary Table 11: The best-fit model (Model 1) for the analysis of the boundaries after the target words, comparing narrow focus to broad focus.

Group-Level Effects:							
~sentence (Number of levels: 24)							
	Estimate	Est.Error	l-95% CI	u-95% CI	Rhat	Bulk_ESS	Tail_ESS
sd(muIP_Intercept)	0.52	0.31	0.03	1.22	1.00	2664	3968
sd(muPW_Intercept)	0.66	0.26	0.17	1.22	1.00	2309	2405
~speaker (Number of levels: 34)							
	Estimate	Est.Error	l-95% CI	u-95% CI	Rhat	Bulk_ESS	Tail_ESS
sd(muIP_Intercept)	1.77	0.53	0.94	3.01	1.00	3142	4383
sd(muPW_Intercept)	1.95	0.41	1.29	2.85	1.00	2966	5273
Population-Level Effects:							
	Estimate	Est.Error	l-95% CI	u-95% CI	Rhat	Bulk_ESS	Tail_ESS
muIP_Intercept	-4.79	0.91	-6.82	-3.23	1.00	4666	4400
muPW_Intercept	-1.26	0.66	-2.61	0.03	1.00	3207	4442
muIP_age_10_11	0.23	1.09	-1.82	2.51	1.00	4788	4603
muIP_age_4_5	0.90	1.18	-1.26	3.46	1.00	4630	5281
muIP_age_7_8	-0.54	1.17	-2.81	1.81	1.00	4745	5253
muIP_broadfocus	1.74	0.51	0.77	2.77	1.00	10890	6165
muPW_age_10_11	-0.49	1.00	-2.50	1.52	1.00	3332	4976
muPW_age_4_5	-1.40	1.16	-3.75	0.91	1.00	4484	4975
muPW_age_7_8	-1.09	1.03	-3.15	0.90	1.00	3662	5104
muPW_broadfocus	-1.74	0.43	-2.63	-0.93	1.00	7491	5885
Exp(B):							
	Estimate	Est.Error	Q2.5	Q97.5			
muIP_Intercept	0.01	2.50	0.00	0.04			
muPW_Intercept	0.28	1.94	0.07	1.04			
muIP_age_10_11	1.26	2.98	0.16	12.28			
muIP_age_4_5	2.46	3.25	0.29	31.72			
muIP_age_7_8	0.58	3.21	0.06	6.13			
muIP_broadfocus	5.68	1.66	2.16	15.99			
muPW_age_10_11	0.61	2.73	0.08	4.58			
muPW_age_4_5	0.25	3.20	0.02	2.49			
muPW_age_7_8	0.34	2.80	0.04	2.46			
muPW_broadfocus	0.18	1.54	0.07	0.40			

Supplementary Table 12: The model containing only FOCUS for the analysis of the boundaries after the target words, comparing narrow focus to broad focus.

Group-Level Effects:							
~sentence (Number of levels: 24)							
	Estimate	Est.Error	l-95% CI	u-95% CI	Rhat	Bulk_ESS	Tail_ESS
sd(muIP_Intercept)	0.48	0.30	0.03	1.16	1.00	1156	1591
sd(muPW_Intercept)	0.63	0.26	0.11	1.18	1.01	813	845
~speaker (Number of levels: 34)							
	Estimate	Est.Error	l-95% CI	u-95% CI	Rhat	Bulk_ESS	Tail_ESS
sd(muIP_Intercept)	1.57	0.43	0.88	2.58	1.00	1524	2371
sd(muPW_Intercept)	1.87	0.37	1.27	2.71	1.00	1339	2064
Population-Level Effects:							
	Estimate	Est.Error	l-95% CI	u-95% CI	Rhat	Bulk_ESS	Tail_ESS
muIP_Intercept	-4.51	0.61	-5.78	-3.43	1.00	2358	2126
muPW_Intercept	-1.81	0.45	-2.76	-0.98	1.00	1324	2034
muIP_broadfocus	1.72	0.50	0.78	2.76	1.00	3854	2769
muPW_broadfocus	-1.70	0.42	-2.54	-0.87	1.00	3309	2672
Exp(B):							
	Estimate	Est.Error	Q2.5	Q97.5			
muIP_Intercept	0.01	1.84	0.00	0.03			
muPW_Intercept	0.16	1.56	0.06	0.37			
muIP_broadfocus	5.58	1.65	2.19	15.77			
muPW_broadfocus	0.18	1.53	0.08	0.42			
